# An Integrated Analysis of Molecular Acclimation to High Light in the Marine Diatom *Phaeodactylum tricornutum*

**DOI:** 10.1371/journal.pone.0007743

**Published:** 2009-11-03

**Authors:** Marianne Nymark, Kristin C. Valle, Tore Brembu, Kasper Hancke, Per Winge, Kjersti Andresen, Geir Johnsen, Atle M. Bones

**Affiliations:** Department of Biology, Norwegian University of Science and Technology, Trondheim, Norway; Umeå Plant Science Centre, Sweden

## Abstract

Photosynthetic diatoms are exposed to rapid and unpredictable changes in irradiance and spectral quality, and must be able to acclimate their light harvesting systems to varying light conditions. Molecular mechanisms behind light acclimation in diatoms are largely unknown. We set out to investigate the mechanisms of high light acclimation in *Phaeodactylum tricornutum* using an integrated approach involving global transcriptional profiling, metabolite profiling and variable fluorescence technique. Algae cultures were acclimated to low light (LL), after which the cultures were transferred to high light (HL). Molecular, metabolic and physiological responses were studied at time points 0.5 h, 3 h, 6 h, 12 h, 24 h and 48 h after transfer to HL conditions. The integrated results indicate that the acclimation mechanisms in diatoms can be divided into an initial response phase (0–0.5 h), an intermediate acclimation phase (3–12 h) and a late acclimation phase (12–48 h). The initial phase is recognized by strong and rapid regulation of genes encoding proteins involved in photosynthesis, pigment metabolism and reactive oxygen species (ROS) scavenging systems. A significant increase in light protecting metabolites occur together with the induction of transcriptional processes involved in protection of cellular structures at this early phase. During the following phases, the metabolite profiling display a pronounced decrease in light harvesting pigments, whereas the variable fluorescence measurements show that the photosynthetic capacity increases strongly during the late acclimation phase. We show that *P. tricornutum* is capable of swift and efficient execution of photoprotective mechanisms, followed by changes in the composition of the photosynthetic machinery that enable the diatoms to utilize the excess energy available in HL. Central molecular players in light protection and acclimation to high irradiance have been identified.

## Introduction

The planktonic diatoms (Bacillariophyceae) account for approximately 40% of the primary production in the world oceans [1]. They are the dominant group of phytoplankton in cold waters [2] and have to cope with highly unpredictable and rapid changes in irradiances (PAR) and spectral quality (E_λ_). In low light it is necessary to collect photons as efficiently as possible, and when the light intensity becomes supersaturating for photosynthesis, it becomes necessary to protect the organism from potential photo-oxidative damage to the photosynthetic machinery. In order to optimize growth and reproduction and to minimize photodamage, phytoplankton has developed a number of mechanisms to modulate the rate of photosynthesis *in situ*. The photoacclimational mechanisms describe the short-term adjustments in response to changing light climate (physiological acclimation), while the photoadaptational mechanisms indicate a long-term evolutionary outcome based on the genes of the given species (genetic adaptation). Both processes work together to maximize evolutionary fitness under a given set of environmental conditions [3]. Over the last four decades, progress in understanding photosynthesis is gradually moving from a descriptive and physiological approach to a molecular one. The whole-genome sequencing of *Thalassiosira pseudonana* [4] and *Phaeodactylum tricornutum* [5] has made possible detailed studies of the genetic basis of the unique properties underlying the ecological and evolutionary success of diatoms. Functional genomics have currently made it possible to investigate the molecular processes behind acclimation to changing environmental conditions in marine organisms. Genomic approaches to this field of investigation are expected to provide new and essential information for studying and monitoring biodiversity, acclimation and adaptations to life in the ocean.

Important and well-known short-term acclimational mechanisms include photochemical quenching (PQ) related to fraction of open reaction centres in PSII [6] and non-photochemical quenching (NPQ) of chlorophyll fluorescence related to pH and photoprotective carotenoids (PPCs), changes in the distribution of excitation energy between photosystems I (PSI) and II (PSII), and damage and repair of PSII [7]. NPQ is the most important short-term “safety valve” that is activated by a sudden increase in irradiance, and can be measured by a decrease in chlorophyll *a* (Chl *a*) fluorescence intensity under HL [8]. In this process, harmful excess energy is dissipated as heat radiation. It is established that NPQ occurs in the light harvesting system of PSII, is triggered by ΔpH and modulated by de-epoxidation of xanthophylls [9]–[11]. In diatoms, the main xanthophyll cycle is the diadinoxanthin (DD) cycle, which involves a forward reaction that converts DD, a carotenoid with low light energy transfer efficiency [12], [13] into diatoxanthin (DT) under conditions of HL. The high photosynthetic flexibility of diatoms is strongly related to their high capacity of NPQ, which can reach a 5-fold higher level than in plants [14]. In addition, the intensity dependence of a rise in variable fluorescence in *P. tricornutum* suggests that light absorbed by the light-harvesting Chl *a*/*c*-Fucoxanthin complex is not preferentially delivered to PSII, but is more equally distributed between the photosystems. These results described by Owens [15] imply that, under both low and high irradiances, adjustments are made in the transfer of excitation energy to the PSII reaction centre which prevents prolonged loss of photosynthetic capacity. These differences in the photosynthetic mechanism of diatoms compared to higher plants may be central to the ecological success of diatoms in a variable light environment. Despite the different photoprotective mechanisms evolved by photosynthetic organisms, light above the saturation point for photosynthesis (the light-saturation index, E_k_) can cause fatal oxidative damage to the PSII reaction centre and result in a decrease in photosynthetic efficiency or photoinhibition [16]–[18]. Photoinhibition occurs when the rate of damage exceeds the capacity of the PSII repair mechanisms [19]. The reaction centre-binding D1 protein is the PSII component most prone to photooxidative damage [20]. The complex mechanisms behind the degradation and repair of PSII and its components have been a subject of investigation for several decades [19], [21]. Damaged D1 proteins must be removed and replaced by newly synthesized molecules for the PSII to recover, and an increased rate of D1 synthesis has been reported for several photosynthetic organisms in conditions of HL [19],[22], [23].

Established long-term acclimation responses to shifts in light conditions include adjustments of the amount and ratios of light harvesting pigments (LHPs) and alterations of the size of the photosynthetic unit (PSU), which are reflected in changes of the maximum photosynthetic capacity of the organism. HL-acclimated cells generally have a low LHP content and a high amount of photoprotective carotenoids; the relationship is inversed for LL-acclimated cells [7], [12], [24], [25]. Some species of phytoplankton acclimate to low irradiances by increasing the size of the PSU [26], defined as the ratio of light-harvesting pigments to P700 reaction centre Chl *a* [27]. Diatoms tend to have large PSU sizes when grown at high, growth-rate-saturating irradiances; in contrast to the smaller units reported for green algae [28] grown at optimal irradiances. When rates of photosynthesis are not limited by light [26], the larger PSU sizes observed for diatoms could represent an evolutionary adaptation to large, daily fluctuating light environments in the ocean. Species with large PSU size growing in HL should respond rapidly when mixed vertically down to low light in deeper water.

Axenically cultured *P. tricornutum* was used to investigate the processes of light acclimation in diatoms. We hypothesised that algae should have a dynamic and fast responding regulatory system that make acclimation to changing light conditions swift and consistent. To study the molecular mechanisms of light acclimation, we performed an integrated analysis combining time series studies of pigment metabolites, fluorometry-based analyses of activity and efficiency of photosystems, and studies of global transcriptional regulation through genome wide transcriptional profiling. The photoprotective carotenoids DD and DT were detected and transcriptional profiles changed dramatically after exposure to HL for only 0.5 h. Pulse Amplitude Modulated (PAM) fluorometry analyses of the photosynthetic capacity showed that significant acclimation to HL conditions were apparent some 12 h after start of the HL treatment. The acclimation processes continued during the next 36 h of exposure to HL conditions. We have identified and categorised transcripts involved in the various phases of light acclimation at a genomic scale.

## Results

To study the mechanisms of protection and acclimation to high irradiances in diatoms, LL acclimated cells were subjected to HL for 0.5 h, 3 h, 6 h, 12 h, 24 h and 48 h. Global transcriptional regulation, change in pigment metabolites and efficiency and capacity of photosynthesis were analyzed in the material harvested from the six time-points. Based on the resulting measurements, the cells seemed to respond to the treatment in three different phases designated the initial response phase (0–0.5 h), the intermediate acclimation phase (3–12 h) and the late acclimation phase (12–48 h).

### Transcriptional profiling of nuclear and plastid transcripts

In addition to the signals from probes representing nuclear-encoded genes, signals from all probes representing chloroplast genes were also detected on the microarrays. This observation indicates that the oligo dT-promoter primer used during the cDNA synthesis step included in the cRNA amplification procedure has been able to hybridize to the poly(A)-rich tail added to endonucleolytically cleaved mature transcripts from chloroplast genes [29]. The poly(A) tail stabilizes nuclear-encoded mRNAs in eukaryotic cells, whereas the poly(A)-rich tail serves as a degradation signal in the chloroplast [29]. The ability to hybridize not only to the poly(A)-tails of the nuclear-encoded mRNAs, but also to the poly(A)-rich tails of the chloroplast-encoded mRNAs has thereby facilitated the generation of cDNA from both types of transcripts. Several chloroplast genes were found to be differentially regulated based on the microarray analyses. If the degradation rates of the chloroplast-encoded mRNAs are the same in cells grown in LL and HL, the expression ratios calculated from probes representing chloroplast genes will be indicative of the regulation of these genes.

To determine the reliability of the microarray data from the chloroplast genes, a two-step qRT-PCR was performed on the RNA material used in the microarray analyses for time points 0.5 h, 3 h, 12 h and 24 h. The relative expression levels of eight chloroplast-encoded genes considered to be of great importance during the photoacclimation process were investigated by qRT-PCR using random primers during the cDNA synthesis. The results showed that relative expression levels obtained from the qRT-PCR analysis correlated well with those produced by the microarray analysis (Supplementary Figure S1). These results imply that the expression ratios obtained from probes representing chloroplast genes actually reflect the relative amounts of the chloroplast gene products.

#### Synthesis of chlorophyll a and steroids

**Figure 1:**
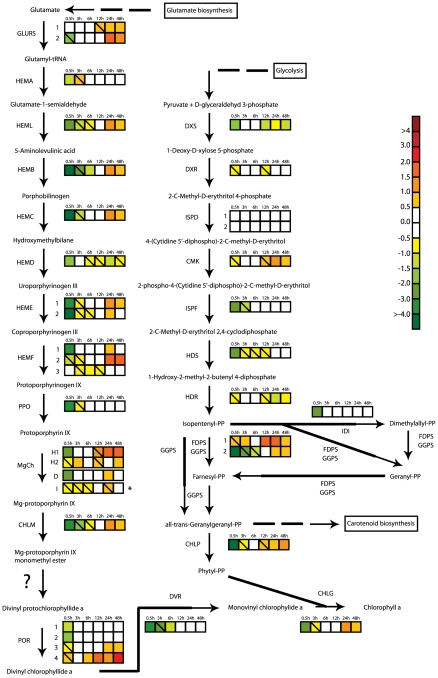
Hypothesized chlorophyll *a* and steroid biosynthetic pathways in *P. tricornutum*. Colored squares indicate the regulation pattern of genes encoding putative enzymes functioning in the two pathways after exposure to HL for 0.5 h, 3 h, 6 h, 12 h, 24 h and 48 h. Squares with a diagonal line inside indicate genes with an expression ratio greater than +/−0.5 that are not significantly regulated. The asterisk marking the expression pattern of subunit I of Mg-chelatase (MgCh) indicates that the gene is chloroplast encoded. The scale on the right represents gene expression ratio values, log_2_ transformed. The gene encoding Mg-protoporphyrin IX monomethyl ester cyclase responsible for converting Mg-protoporphyrin IX monomethyl ester to divinyl protochlorophyllide *a* in higher plants [30] is absent in *P. tricornutum*, and this step is marked with a question mark in the figure. The abbreviations used are: GLURS: glutamyl-tRNA synthetase; HEMA: glutamyl-tRNA reductase; HEML: glutamate-1-semialdehyde 2,1-aminomutase, HEMB: porphobilinogen synthase; HEMC: hydroxymethylbilane synthase; HEMD: uroporphyrinogen-III synthase; HEME: uroporphyrinogen decarboxylase; HEMF: coproporphyrinogen III oxidase; PPO: protoporphyrinogen oxidase; MgCh: magnesium chelatase; CHLM: Mg-protoporphyrin IX methyl transferase; POR: protochlorophyllide oxidoreductase; DVR: divinyl protochlorophyllide a 8-vinyl reductase; CHLG: chlorophyll synthase; DXS: deoxyxylulose-5-phosphate synthase; DXR: 1-deoxy-D-xylulose 5-phosphate reductoisomerase; ISPD: 2-C-methyl-D-erythritol 4-phosphate cytidylyltransferase; CMK: 4-diphosphocytidyl-2-C-methyl-D-erythritol kinase; ISPF: 2-C-methyl-D-erythritol 2,4-cyclodiphosphate synthase; HDS: 1-hydroxy-2-methyl-2-(E)-butenyl 4-diphosphate synthase; HDR: 4-hydroxy-3-methylbut-2-enyl diphosphate reductase; FDPS: farnesyl diphosphate synthase; GGPS: geranylgeranyl pyrophosphate synthase; IDI: isopentenyl pyrophosphate:dimethylallyl pyrophosphate isomerise; CHLP: geranylgeranyl reductase.

An immediate response after transfer to HL conditions was a dramatic reduction in expression of transcripts encoding enzymes in the Chl *a* biosynthesis. In higher plants, Chl *a* is synthesized from glutamate in a 15 step biosynthetic pathway [30] through the cooperative action of a range of different enzymes. Transcripts for all genes encoding enzymes involved in the Chl *a* synthesis of higher plants, except for the gene encoding Mg-protoporphyrin IX monomethyl ester cyclase (MgCy), were identified in *P. tricornutum* (Figure 1). MgCy is also absent in *Thalassiosira pseudonana* [31]. In higher plants, MgCy is responsible for converting Mg-protoporphyrin IX monomethyl ester to divinyl protochlorophyllide. The majority of the enzymes involved in the Chl *a* biosynthetic pathway are represented in the *P. tricornutum* genome by a single gene. The enzymes encoded by multi-gene families are glutamyl-tRNA synthetase (two genes, *GLURS_1-2*), uroporphyrinogen decarboxylase(*HEME_1-2*), coproporphyrinogen III oxidase (*HEMF_1-3*), protochlorophyllide oxidoreductase (*POR_1-4*), and the H subunit (*CHLH_1-2*) of Magnesium-chelatase (MgCh). All genes encoding enzymes in the Chl *a* pathway are found in the nucleus, with the exception of the *CHLI* gene encoding the MgCh I subunit, which is chloroplast-encoded. The expression of all nuclear single copy genes and at least one of each type of the nuclear-encoded multi-copy genes involved in Chl *a* biosynthesis dropped dramatically after 0.5 h (Figure 1), indicating a strong down-regulation of every step in the synthesis of Chl *a* at this initial response phase. The gene encoding CHLI was not significantly affected by the HL treatment. Phytyl diphosphate produced by the steroid biosynthesis pathway functions together with monovinyl chlorophyllide *a* from the Chl *a* biosynthesis pathway as a substrate for chlorophyll synthetase in the last step in the formation of Chl *a*. The genes encoding putative geranylgeranyl diphosphate synthase (GGPS) and geranylgeranyl reductase (CHLP), responsible for converting isopentenyl diphosphate through several possible intermediates to phytyl diphosphate, were also strongly down-regulated at the initial phase (Figure 1). During the intermediate acclimation phase, the transcript levels of the above mentioned nuclear encoded genes gradually recovered, and after 12 h most genes were back to LL levels. The majority of these genes were found to be moderately up-regulated in the late acclimation phase.

#### Synthesis of carotenoids

**Figure 2:**
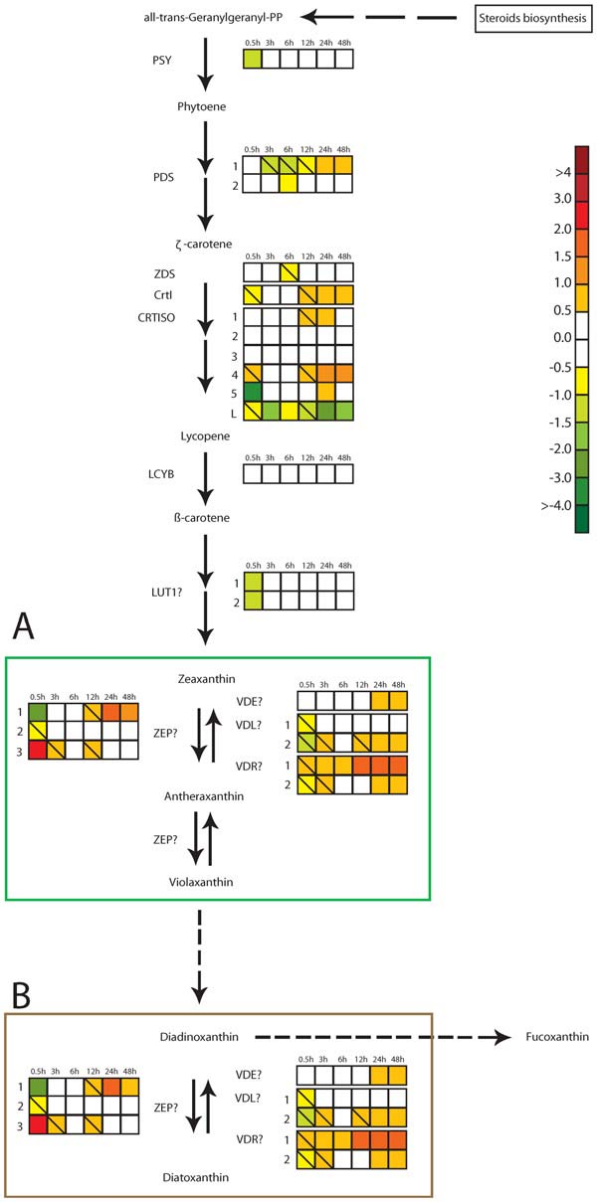
Hypothesized carotenoid biosynthetic pathway in *P. tricornutum* according to Coesel *et al*. [**32**] **.** Colored squares indicate the regulation pattern of genes encoding putative enzymes involved in the synthesis of carotenoids after exposure to HL for 0.5 h, 3 h, 6 h, 12 h, 24 h and 48 h. Squares with a diagonal line inside indicate genes with an expression ratio (log_2_ transformed) greater than +/−0.5 that are not significantly regulated. The scale on the right represents gene expression ratio values, log_2_ transformed. The violaxanthin cycle (A) and the diadinoxanthin cycle (B) are boxed. Dashed arrows indicate the hypothetical conversion of violaxanthin to diadinoxanthin and the formation of fucoxanthin from diadinoxanthin, as proposed by Lohr and Wilhelm [52], [53]. The abbreviations used are PSY: phytoene synthase; PDS: phytoene desaturase; ZDS: ζ-carotene desaturase, CRTISO: carotenoid isomerase; crtI: bacterial-like desaturase; LCYB: lycopene β-cyclase; LUT: lutein deficient-like; ZEP: zeaxanthin epoxidase; VDE: violaxanthin de de-epoxidase; VDL: violaxanthin de de-epoxidase-like; VDR: violaxanthin de de-epoxidase related.

A hypothetical carotenoid biosynthetic pathway is presented in Figure 2, according to Coesel *et al*. [32]. The genes identified and proposed to be involved in the synthesis of carotenoids in *P. tricornutum* by Coesel *et al*. are indicated on the figure. With a few intriguing exceptions the transcript levels of these genes were in general little affected by the exposure to HL. The most interesting and specific responses were the immediate regulation of genes that might be involved in controlling the forward reactions of the two xanthophyll cycles in *P. tricornutum*. The *P. tricornutum* genome has been found to contain three genes encoding zeaxanthin epoxidase (ZEP1-3), but it is not known whether these genes encode enzymes involved in the violaxanthin cycle, the diadinoxanthin cycle or possible both cycles. After exposure to HL for 0.5 h, the expression level of *ZEP1* was clearly down-regulated, while *ZEP3* gene expression was clearly up-regulated, indicating that these two genes encode enzymes with different functions. One possibility is that the *ZEP1* gene encodes the enzyme responsible for converting zeaxanthin to violaxanthin in LL, while the *ZEP3* gene encodes the enzyme that converts DD to DT in HL. *ZEP1* and *ZEP3* both showed little or no regulation during the intermediate phase. At the late acclimation phase, the *ZEP1* gene expression was moderately increased, while the *ZEP3* gene expression level was maintained at LL levels.

#### Light harvesting antenna proteins

**Figure 3:**
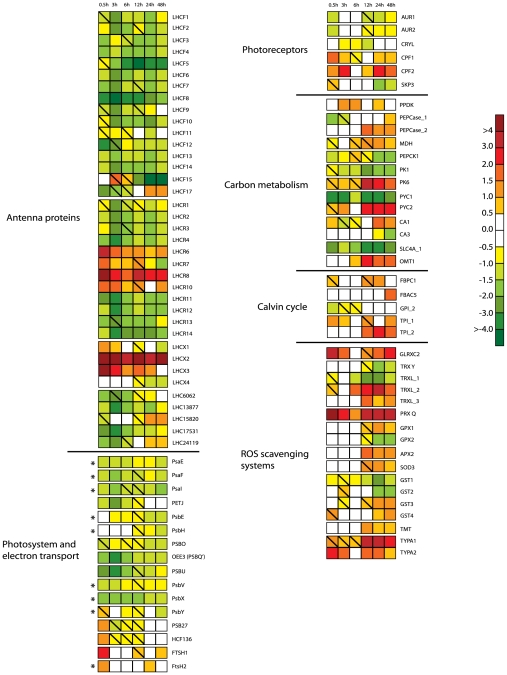
Regulation pattern of HL affected genes during the acclimation period. The differentially regulated genes encode proteins involved in light sensing, antenna proteins, photoreceptors, components involved in oxidative photosynthesis, carbon metabolism, Calvin cycle and ROS scavenging systems after exposure to HL for 0.5 h, 3 h, 6 h, 12 h, 24 h and 48 h. The color code indicates expression values. Squares with a diagonal line inside indicate genes with an expression ratio (log_2_ transformed) greater than +/−0.5 that are not significantly regulated. Genes where at least one of the probes representing the genes were significantly regulated by >2-fold at least at one time point during the acclimation period, were included in the figure. The expression patterns of genes marked with an asterisk are chloroplast encoded. The scale on the right represents gene expression ratio values, log_2_ transformed. The abbreviations used are LHCF: major fucoxanthin Chl *a/c* proteins; LHCR: red algal-like proteins; LHCX: LI818-like proteins; LHC#: unclassified light harvesting proteins; Psa: PSI proteins; PETJ: cytochrome c6; Psb: PSII proteins; HCF: high Chl fluorescence; FtsH: Filamentation temperature sensitive H; AUR: aureochrome; CRYL: cryptochrome-like protein; CPF: cryptochrome; SKP3: Sensor Kinase Protein 3; PPDK: pyruvate-phosphate dikinase; PEPCase: phosphoenolpyruvate carboxylase; MDH: malate dehydrogenase; PEPCK: phosphoenolpyruvate carboxykinase; PK: pyruvate kinase; PYC: pyruvate carboxylase; CA: carbonic anhydrase; SLC4A: bicarbonate transporter; OMT: oxoglutarate/malate transporter; FBPC: fructose-1,6-bisphosphatase; FBAC: fructose-1,6-bisphosphate aldolase; GPI: glucose-6-phosphate isomerase; TPI: triosephosphate isomerase; GLRXC: glutaredoxin; TRX: thioredoxin; TRXLl: thioredoxin-like; PRX: peroxiredoxin; GPX: glutathione peroxidase; APX: ascorbate peroxidise; SOD: superoxide dismutase; GST: glutathione S-transferase; TMT: gamma-tocopherol methyltransferase; TYPA: tyrosine phosphorylation protein A.

The *P. tricornutum* genome is predicted to encode at least 40 genes belonging to the light-harvesting complex (LHC) superfamily. These gene transcripts were all detected by the whole-genome array, and 37 out of the 40 genes were found to be significantly regulated at least at one time point (Figure 3). The diatom light harvesting genes are divided into three main groups [33]: the *LHCF*'s, encoding the major fucoxanthin Chl *a*/*c* proteins, the red algal-like *LHCR*'s and the LI818-like *LHCX*'s.

From the results presented in Figure 3 it is evident that most of the transcripts encoding putative light harvesting antenna proteins are continuously down-regulated during all three phases, reflecting the expected acclimation to higher light irradiances. However, a handful of transcripts in the same families are strongly up-regulated, especially at the initial phase, indicating a role in photoprotection. The most strongly induced genes, *LHCR6*, *LHCR8*, *LHCX2* and *LHCX3*, increased dramatically after exposure to HL and were up-regulated as much as 13-36 times already after 0.5 h. The expression of the *LHCX2* gene remained at almost the same high level during the entire length of the experiment, while the expression of *LHCR6* and *LHCR8* dropped during the intermediate phase and stabilized at a lower level. The *LHCX3* transcript level decreased gradually with time and was back to LL levels at the late acclimation phase.

#### Photosystems and electron transport chain

Oxidative photosynthesis is catalyzed by the four multi-subunit complexes PSI, PSII, the cytochrome b6f complex and F-ATPase [34]. In *P. tricornutum* the vast majority of the genes encoding subunits of the mentioned membrane-protein complexes are localized to the chloroplast genome [35], while some genes have been transferred to the nucleus. The transcript levels of eight genes localized to the chloroplast genome were analyzed by qRT-PCR, including two PSII genes (*psbA* and *psbV*), two PSI genes (*psaA* and *psaE*), and an ATP-dependent metalloprotease (*ftsH_2*). Both chloroplast- and nuclear-encoded genes representing proteins involved in photosynthesis and repair of photo-damaged PSII were differentially expressed during the acclimation period, as indicated on Figure 3.

The nuclear-encoded genes *PSBO*, *OEE3* (*PSBQ'*) and *PSBU* and the chloroplast encoded *psbV*, encoding putative subunits of the oxygen evolving complex of PSII [36], were all found to be constitutively repressed under HL conditions (Figure 3). This was also evident for the nuclear-encoded *PSBM* gene, encoding one of the small transmembrane proteins of the PSII reaction center [36]. The microarray data based on signals from polyadenylated chloroplast transcripts also indicated that PSII genes like *psbE*, *psbH*, *psbY* and *psbX* were down-regulated as a response to the HL treatment during some or all of the acclimation phases. These genes are all predicted to encode small transmembrane proteins located in the reaction center of PSII [36]. In addition, a conserved open-reading frame named *ycf66* [35], positioned in a gene cluster between the chloroplast-encoded *psbV* and *psbX* genes, displayed the same regulation pattern as the two *psb* genes, being down-regulated at all times (data not shown). Two genes assumed to encode the *PSB27* and the *HCF136* proteins that function in assembly and repair of PSII in both chloroplasts [37], [38] and cyanobacteria [39,40) showed an increase in expression level at the initial response phase. During the intermediate phase, the *PSB27* and *HCF136* genes both displayed a slight down-regulation, whereas the expression levels at the late acclimation phase was back to LL levels. Two genes encoding ATP-dependent metalloproteases (FTSH1 and FTSH2) found to be involved in the degradation of photodamaged D1 protein in plants and cyanobacteria [21], were also significantly induced immediately after transfer to HL. At the two latest phases of the acclimation period the *FTSH* genes were unaffected or slightly up-regulated. The *psbA* and *psbD* genes, encoding the D1 and D2 proteins that together form the core of the PSII reaction center [34], showed no significant response to the HL treatment. This heterodimer binds several cofactors, including chlorophylls, pheophytin a molecules and the plastoquinones Q_A_ and Q_B_ involved in the electron transfer in PSII.

Among the chloroplast-localized *psa* genes encoding subunits of PSI, the *psaE* gene was confirmed by qRT-PCR to be continuously repressed when subjected to HL. PsaE is one of the subunits of the PSI core complex and functions as a binding site for soluble ferredoxin, and is also involved in cyclic electron transport [41]. The microarray data indicated that the expression of the *psaF* and *psaI* genes, encoding two additional core proteins, was also down-regulated in the HL cultures. In higher plants, the PsaF protein binds the electron donor plastocyanin and an antenna protein dimer, whereas PsaI stabilizes another core subunit [41].

#### Photoreceptors

Genes predicted to encode photoreceptors like blue-light sensing aureochromes and cryptochromes, and red/far-red perceiving phytochromes have been identified in *P. tricornutum* [5]. As indicated on Figure 3, most of the regulated photoreceptor genes displayed a moderate response to the HL treatment, with the exception of one of the cryptochromes (*CPF2*). *CPF2* expression levels increased up to 4–5 fold in HL compared to LL cultures cells at the beginning of the intermediate phase and also during the late acclimation phase.

#### Carbon metabolism and Calvin cycle

Kroth *et al.* [42] has identified 16 genes putatively involved in a C4-like photosynthesis in *P. tricornutum*. The protein products of these genes were predicted to be localized to three different cell compartments; endoplasmatic reticulum (ER), mitochondria and plastid. In our experiment, 13 of the mentioned genes were found to be significantly regulated by HL at one or several time points (Figure 3). Among the most pronounced down-regulated genes were *PYC1*, encoding a mitochondrial-localized pyruvate carboxylase, and *SLC4A_1*, encoding a bicarbonate transporter predicted to be localized to the plastid [42]. *PYC1* and *SLC4A_1* displayed a decrease in expression levels during the entire acclimation period as a response to the HL treatment. The gene encoding phosphoenolpyruvate carboxylase (PEPCase_2), predicted to be targeted to the ER, mitochondrial-localized malate dehydrogenase (MDH), a pyruvate kinase (PK6), an oxoglutarate/malate transporter (OMT1), and the chloroplast-targeted pyruvate carboxylase (PYC2) all showed a similar regulation pattern after being subjected to HL conditions. These genes were all unaffected or only slightly regulated during the first hours of HL exposure, and up-regulated 2–11 times at the late acclimation phase.

Kroth *et al.* [42] has also identified 26 genes possibly encoding Calvin cycle enzymes, which are predicted to be distributed between the plastid, mitochondria and the cytosol. These genes, including the chloroplast-encoded subunits of ribulose-bisphosphate carboxylase oxygenase (RUBISCO), showed in general little or no response to the HL treatment. A gene presumed to encode a triosephosphate isomerase (TPI_2) predicted to be targeted to the plastid, was among the most regulated Calvin cycle genes. The *TPI_2* gene displayed a 3–6 fold up-regulation in HL at the late acclimation phase (Figure 3).

#### ROS scavenging systems

Reactive oxygen species (ROS) form as by-products during photosynthesis and can result in significant damage to cellular components [43]. Genes encoding known enzymatic antioxidants like catalases, superoxide dismutases (SOD), ascorbate peroxidases (APX), gluthatione S-transferases (GST) and glutathione peroxidases (GPX) were unaffected, slightly up-regulated or even down-regulated during the two first response and acclimation phases (Figure 3). At the late acclimation phase, only a few of these genes showed a significant increase in expression levels. *SOD3*, encoding a CuZn superoxide dismutase, *APX2*, *GPX1*, *GST3* and *GST4* displayed a 2–3 fold up-regulation at these time points. A gene encoding a putative gamma-tocopherol methyltransferase (TMT) responsible for converting gamma-tocopherol to the non-enzymatic antioxidant alpha-tocopherol [44] was also moderately up-regulated at the latest time points.

In contrast, a gene similar to the antioxidant peroxiredoxin Q (PRX Q) in *Arabidopsis thaliana* was among the strongest and most consistent up-regulated genes of the entire data set. *PRX Q* showed an immediate response to the HL treatment and was up-regulated as much as 24 times after just 0.5 h in HL. During the intermediate phase the expression levels dropped, but increased again after 12 hours and stayed high at the late acclimation phase. The *A. thaliana* PRX Q is targeted to the plastid and has been suggested to be involved in protection of ROS generated in photosynthesis [45]. PRX Q reduces H_2_O_2_ using a thiol and is reactivated by thioredoxins. Other peroxiredoxins have been shown to be reactivated by glutaredoxins [46]. Genes encoding a glutaredoxin (GLRXC2), a thioredoxin (TRX Y) and several genes possibly encoding thioredoxin-like (TRXL_1-3) proteins were differentially regulated as a response to HL. *GLRXC2* are predicted to be targeted to the plastid [42], and showed a similar regulation pattern as *PRX Q*. *GLRXC2* displayed a 20-fold increase in expression level at the initial response phase, a drop in the expression level during the next hours and a subsequent increase at the late acclimation phase. *TRXL_2* and *TRXL_3* were also up-regulated at several time points. TRX Y, predicted to be plastid localized [42], and TRXL_1 were down-regulated and might be involved in enzyme regulation [47] instead of stress response.

Based on experiments with the halophytic plant *Suaeda salsa,* Wang *et al.* [48] has suggested that a member of the TypA/BipA GTPase family named SsTypA1 might play a crucial role in the defence against ROS damage, possibly functioning as a translational regulator of the stress-responsive proteins involved in ROS scavenging in chloroplast. Two genes identified in *P. tricornutum* (designated *TypA1-2*) encoding putative TypA GTP-binding proteins were up-regulated for all, or all but one time-point during the HL experiment. The transcriptional analyses of the *TypA* genes revealed that the expression of the *TypA1* gene reached its maximum level during the late acclimation phase, where the transcription was up-regulated almost 15 times in the HL-cultures compared to the LL-cultures, whereas the *TypA2* gene peaked after only 0.5 h in HL (Figure 3).

### Pigment analysis

The pigmentation in *P. tricornutum* comprises the major light harvesting pigments (LHPs) Chl *a*, Chl c_1_ and c_2_, Fucoxanthin (Fuco), the photoprotective carotenoid diadinoxanthin (DD) which can be de-epoxidized to diatoxanthin (DT) in addition to the ubiquitous β -carotene found in all photosynthetic organisms [49]. As expected, the HPLC analyses showed that Chls *a* and *c*, Fuco, DD and DT (the latter not found in the LL cultures) were the dominating pigments in the algae. Violaxanthin, ββ-carotene and a few derivatives of Chl *a* and Fuco were present in trace amounts.

#### Chlorophyll and Fucoxanthin

**Figure 4:**
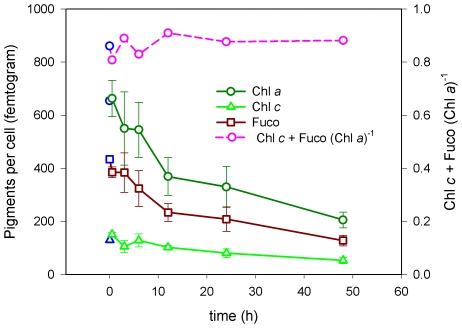
Main [LHPs] per cell and their ratio as a function of HL exposure time. The Chl *a*, Chl *c* and Fuco concentrations per cell and the ratio of Fuco plus Chl *c* to Chl *a* as a function of high-light (500 µmol m^−2^s^−1^) exposure time. Incubation was performed in a LL (35 µmol m^−2^s^−1^) exponentially growing 10L batch culture 3 weeks prior to HL exposure. The 0 h sample value is the mean of the 18 LL control samples (blue symbol). HL exposure values are the mean of three biological parallels. Values are presented with±SD bars.

The concentration of Chl *a* per cell ([Chl *a*]) remained unchanged during the initial response phase, after which [Chl *a*] decreased gradually during the next two acclimation phases (Figure 4). Although present in much lower concentrations, the measurements of Chl *c* per cell ([Chl *c*]) showed a similar pattern. Concentrations of Fuco per cell ([Fuco]) showed an immediate decline from the onset of HL and throughout the experiment. The ratio between the LHPs [Fuco+Chl *c*] and [Chl *a*] was stable during the experiment, ranging between 0.8 and 0.9. These results imply a highly effective acclimation to changed light climate, as the concentration of LHPs are down-regulated to adjust to the Chl *a* concentration.

#### Diadinoxanthin, diatoxanthin and NPQ

**Figure 5:**
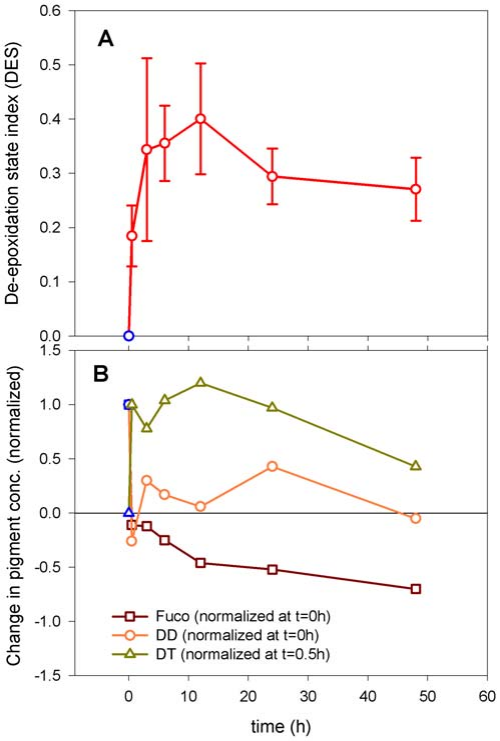
De-epoxidation state index (DES) and change in [Fuco], [DD] and [DT] per cell. A) De-epoxidation state index (DES) index calculated from the HPLC pigment data. The 0 h sample value is the mean of the 18 LL control samples (blue symbol). HL exposure values are the mean of three biological parallels. Incubations as in Figure 4. Values are presented with±SD bars. (B) Change in Fucoxanthin, Diadinoxanthin and Diatoxanthin cell concentration as a function of high light exposure time. Change in pigment concentration for Fuco (normalized to LL, t = 0 h), DD (as for Fuco) and DT (normalized to HL at 0.5 h) as a function of time after HL exposure. Values are average of three parallel HPLC samples. Incubations as in Figure 4.

0.5 h after HL exposure of LL acclimated cells, de-epoxidation of DD to form DT had started to take place, thereby facilitating the dissipation of excess light energy by NPQ. The increasing de-epoxidation state (DES) index during the initial and intermediate acclimation phases describes the rapid conversion of DD to DT (Figure 5A). At the shift from the intermediate to the late acclimation phase there was a decrease in DES, after which the algae seemed to have acclimated to the increased irradiance, reflected by the small decrease in DES from 24 to 48 h. The changes in the fractions of cellular DD, DT and Fuco from the original LL pool sizes as a function of HL exposure time is shown in Figure 5B. From the decrease in the DD fraction and the increase of the DT fraction measured in cultures that had been subjected to HL for 0.5 h, it is evident that these two pigments are in an inverse relationship with each other (Figure 5B). In the intermediate acclimation face the 3 h HL exposure time resulted in increased production of DD as a response to an increased need for photoprotection. From 3 h to 12 h the cellular [DD] pool size decreased while cellular [DT], from de-epoxidation of DD, increased simultaneously. The late acclimation phase showed a new peak in cellular DD after 24 h, indicating cell division. As the algae acclimated to HL (48 h) [DT] per cell was reduced. From onset of HL the cellular Fuco fraction started to decrease, and continued to do so during the experimental period. This suggests that DD might be the precursor of both DT and Fuco.

### Variable Chl fluorescence

**Figure 6:**
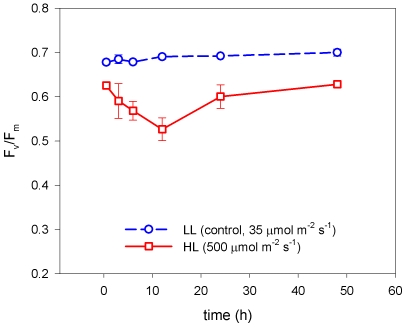
The maximum quantum yield after HL exposure. The maximum quantum yield (Fv/Fm) as a function of time after exposure to HL (500 µmol m^−2^ s^−1^, red squares, solid line) and LL (35 µmolm^−2^ s^−1^, blue circles, dashed line) measured using variable fluorescence (PAM) after keeping the samples for 3 min in the dark. Bars are S.D. (n = 3).

The Chl *a* variable fluorescence illustrate the overall physiological response of *P. tricornutum,* and the data can be read as a proxy for the photosynthetic efficiency and capacity of the cell. The photosynthetic (PSII) efficiency, measured from the maximum quantum yield, F_v_/F_m_, showed a decrease in the initial and intermediate phase (<12 h) after exposure to HL, illustrating that the ratio of electrons generated in PSII to photons absorbed by light-harvesting pigments decrease (Figure 6). During the late acclimation phase (>12 h), F_v_/F_m_ increased again to a level similar to the initial phase. The HL treated culture overall showed a lower F_v_/F_m_ than the LL samples.

**Figure 7:**
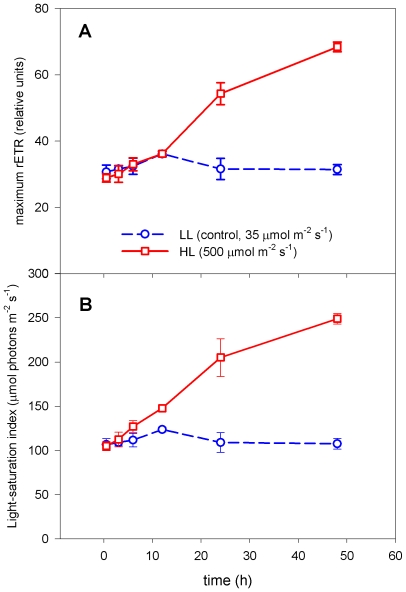
The photosynthetic capacity and the light-saturation index. The A) maximum photosynthetic capacity ( i.e. maximum light-saturated rETR) and B) the light-saturation index *versus* time after exposure to HL (500 µmol m^−2^ s^−1^, red squares, solid line) and LL (35 µmol m^−2^ s^−1^, blue circles, dashed line), calculated from the photosynthesis *vs.* irradiance relationship measured using variable Chl *a* fluorescence (PAM). Bars are S.D. (n = 3).

The maximum photosynthetic capacity (i.e. the maximum relative light-saturated electron transfer rate, rETR) calculated from the P *vs.* E relationship was not significantly altered by HL treatment during the initial and intermediate phase; however, it markedly increased during the late acclimation phase (>12 h) compared to the control (LL treatment, Figure 7A). The maximum rETR express the maximum amount of electrons generated in PSII that is available to the ATP and NADPH_2_ synthesis at ambient light, and thus is an estimate of the maximum photosynthetic capacity. At 48 h, the maximum rETR had increased >2 fold in the HL treatment culture compared to the LL acclimated culture.

The light-saturation index, which is an indicator for the photo-acclimation status of the cell, showed a linear increasing trend as a function of time after HL exposure (Figure 7B). Data indicated that the physiological acclimation status of *P. tricornutum* increased from the initial phase throughout the late acclimation phase, with no sign of reaching an upper limit within the investigated 48 h time frame. The light saturation index is a proxy for the irradiance that is needed to saturate photosynthesis, i.e. the threshold irradiance that separates light-limited and light-saturated photosynthesis.

## Discussion

In this study, we set out to investigate the mechanisms behind photo-acclimation to high-light conditions in diatoms by combining molecular and physiological methods. Physiological methods have been utilized for several decades to investigate how diatoms process the sudden changes in irradiance that they are exposed to in nature. Small scale analyses of photoregulated gene expression have previously been performed [33], [50], [51]. However, this is the first time global transcriptional profiling has been utilized to investigate these mechanisms in a diatom. The results show that exposure to HL results in dramatic changes in the transcriptional profiles. The measurements indicated that the responses could be divided into three distinct phases; an initial response phase (0–0.5 h), an intermediate acclimation phase (3–12 h) and a late acclimation phase (12–48 h), which will be discussed below. A summary of the most important processes in protection and acclimation to HL in *Phaeodactylum tricornutum* is given in Table 1.

**Table 1:** Summary of the most important processes in protection and acclimation to high light (HL) in *P. tricornutum*.

Level		Initial response phase	Intermediate acclimation phase	Late acclimation phase
**Molecular**	Genes encoding antenna proteins possibly involved in photoprotection	**+ + +**	**+ +**	**+ +**
	Genes encoding proteins involved in degradation, assembly and repair of PSII components	**+**		
	Genes encoding specific proteins involved in ROS scavenging	**+ + +**	**+**	**+ +**
	Genes encoding additional proteins involved in ROS scavenging			**+**
	Zeaxanthin epoxidase gene possibly encoding the enzyme responsible for conversion of DD to DT	**+ +**		
	Genes encoding enzymes involved in Chl *a* biosynthesis	**÷ ÷ ÷**		**+**
	Genes encoding components of the light harvesting and photosynthetic apparatus	**÷**	**÷ ÷**	**÷**
**Metabolic**	DES index	**+**	**+ + +**	**+ +**
	Fuco content per cell	**÷**	**÷ ÷**	**÷ ÷ ÷**
	Chl *a* content per cell		**÷ ÷**	**÷ ÷ ÷**
**Physiological**	Photosynthetic efficiency and capacity			**+ +**
**Summary**	Photoprotection mechanisms	Execution of photoprotection mechanisms	Photoprotection mechanisms still active, but not as pronounced as in the early phase	Photoprotection mechanisms still active, but not as pronounced as in the early phase
	HL acclimation processes	HL acclimation processes initiated at the transcriptional level.	HL acclimation processes observed at the transcriptional and metabolic level, but not at the physiological level	HL acclimation processes observed at all levels. Changes in the composition of the photosynthetic apparatus enable the diatoms to perform highly efficient photosynthesis in HL

Plus (+) and minus (÷) symbols indicate an increase or decrease in HL compared to LL levels. The abbreviations used are PSII: photosystem II; ROS: reactive oxygen species; DD: diadinoxanthin; DT: diatoxanthin; Chl: chlorophyll; DES de-epoxidation state; Fuco: fucoxanthin.

### The initial response phase

At the molecular level, the initial response phase is characterized by a fast and strong regulation of several genes encoding members of the light harvesting machinery and a few components of the ROS scavenging systems. One striking feature of this initial phase is a severe repression of nuclear-encoded genes involved in all steps of the Chl *a* metabolism, suggesting a light dependent and synchronised regulation of the transcription of these genes (Figure 1). The regulation of Chl *a* biosynthesis is only partly understood in plants, and it is generally believed that the regulation of enzyme activity is more important than a transcriptional regulation in terms of controlling the amount of Chl *a* produced [30]. Chl *a* and most intermediate molecules of the Chl *a* synthesis produce ROS under illumination if present in excess amounts [30], and it is therefore critical to accurately control the levels of these compounds. The down-regulation of Chl *a* metabolism at the transcriptional level was not reflected by the measurements of Chl *a* concentration at this early stage (Figure 4).

NPQ is the most important short-term photoprotection mechanism in diatoms, where it involves the conversion of DD into DT under conditions of HL [14] and is recognized by the increasing DES index at this initial response phase. In our study, a marked decrease of DD and an increase of DT occurred together with a decrease in Fuco content after 0.5 h in HL (Figure 5B), supporting earlier suggestions that DD is also a precursor of Fuco [52], [53]. In this way, the conversion of DD to DT not only leads to dissipation of excess energy, but the decrease in cellular [Fuco] also suppresses light harvesting and thereby light energy transfer for photosynthesis. The *P. tricornutum* genome contains three candidate genes that might encode the enzyme responsible for the conversion of DD to DT in HL. Based on the specific up-regulation seen only for the *ZEP3* gene at this early stage, we suggest that the product from this gene performs the de-epoxidation reaction (Figure 2). In higher plants, a PSII protein, PsbS, is important for NPQ [8]. No homolog for this gene has been identified in diatoms [4], [5], but one or several of the antenna proteins classified as LHCX's might serve the role of PsbS in photoprotecion [33]. The LHCX's are the diatom homologs of the LI818 proteins first identified in *Chlamydomonas* [54]. Members of this group of proteins are known to be induced in response to HL stress and are suggested to have photoprotective properties [33], [50], [55]. The transcription of the *P. tricornutum LHCX2* and *LHCX3* genes were found to be strongly induced after only 0.5 h in HL, suggesting that there might be a connection between these results and the other mechanisms involved in NPQ in diatoms (Figure 3). The expression level of both *LHCX'*s peaked at this initial response phase. The transcription of the *LHCX2* gene stayed high throughout the experiment, whereas the *LHCX3* expression decreased with time. The fast and drastic up-regulation of two of the *LHCX's* and also two of the *LHCR's* at this early time-point are in sharp contrast to the decrease in transcription level observed for almost all other genes encoding diatom antenna proteins, implying different roles for these proteins in protection and acclimation to HL (Figure 3).

Although photosynthetic organisms are in the possession of numerous light sensing and acclimation mechanisms [56], excess light can generate ROS, causing damage especially to the PSII, where the D1 core protein is particularly vulnerable [20]. Excess light usually leads to an enhanced synthesis of D1 protein to replace the damaged ones [19]. In our study, several genes encoding subunits of PSII was continuously down-regulated as a response to HL, whereas the *psbA* gene encoding the D1 protein was maintained at LL levels, indicating a greater demand for this PSII transcript. In green algae the increase in D1 protein synthesis far exceeded the accumulation of the corresponding mRNA [22], suggesting that the D1 protein synthesis might also be up-regulated by HL in our study despite the lack of regulation at the transcriptional level. The early phase specific up-regulation of the two *ftsH* genes encoding proteases functioning in degradation of photodamaged D1 protein [21], and also of two genes (*HCF136* and *PSB27*) encoding proteins involved in assembly and repair of PSII [37]–[40] implies that the HL treatment has caused photodamage to the complex and that mechanisms necessary for the PSII to recover have been executed. Another protection mechanism initiated in the HL subjected cells is the severe induction of the H_2_O_2_ peroxidase gene *PRX Q*, encoding a protein similar to the *A. thaliana* peroxiredoxin Q that is suggested to be involved in protection of PSII [45]. The up-regulation of the *PRX Q* gene was accompanied with an almost equally strong increase in the transcription level of a glutaredoxin (GLRXC2) predicted to be targeted to the chloroplast [42]. The expression of *PRX Q* and *GLRXC2* displays a similar regulation pattern throughout the entire length of the experiment. This observation indicates that the diatom PRX Q might be reduced and thereby reactivated by glutaredoxin instead of thioredoxins, which is the case in *A. thaliana* [45].

At the initial response phase, neither the maximum rETR (Figure 7A) nor the light-saturation index (Figure 7B) changed significantly in the HL exposed cultures compared to the LL cultures. This shows that the *P. tricornutum* cells are able to maintain their photosynthetic efficiency despite the HL stress and that their capability to utilise incoming photons remain unchanged.

### The intermediate acclimation phase

The global transcriptional profiling revealed a strong and rapid response to the change in irradiance already after 0.5 h, whereas most physiological responses first became apparent at the intermediate acclimation phase (Figure 7A and B). This phase is characterised by a marked decline in cellular LHPs, where the decrease in Fuco levels observed after 0.5 h in HL continues, and is now also followed by a significant fall in Chl *a* level. The expression of the genes involved in the synthesis of Chl *a* return to LL levels during this period (Figure 1). This observation suggests that the reduction of the amount of Chl *a* might be caused not only by the down-regulation at the transcriptional level seen at the initial response phase, but also by other mechanisms like repression of the corresponding enzyme activity [30] and regulation of Chl *a* degradation. The decrease in LHPs also lowers the need for the antenna proteins that bind Chls and Fuco and thereby anchor them to the thylakoid membranes. This is reflected at the transcriptional level by the repression of most genes encoding antenna proteins seen throughout the experiment (Figure 3). Down-regulation of members of the LHC family have also been observed in several other HL experiments with diatoms, green algae and higher plants [33], [56], [57]. Several of the continuously repressed genes involved in the light harvesting machinery, including components of the photosystems, display the strongest down-regulation at the beginning of this phase.

The continuous up-regulation of the *LHCX's* that might function in the role of *psbS* and the still increasing DES index implies that the diatoms continues to dissipate the excess light energy as heat dissipation by NPQ at this intermediate phase.

### The late acclimation phase

During the first 12 hours of the HL treatment, the diatom cells seem to protect and adjust the photosynthetic apparatus to the new light regime without being able to make use of the increased amounts of light energy available for photosynthesis and growth. After 12 hours in HL, the acclimation mechanisms observed at the molecular level are replaced or supplemented with changes supporting the shorter generation time (data not shown) and thereby an accelerated protein synthesis, and responses characteristic for the late acclimation phase. The characteristic responses include an increase of the transcript levels of genes involved in the removal of potentially harmful ROS (Figure 3), in particular a strong induction of *TypA1* encoding a member of the TypA/BipA GTPase family suggested to function as a translational regulator of stress-responsive proteins involved in ROS scavenging in chloroplast [48]. ROS are inevitable by-products of photosynthesis, and the increased demand of antioxidants might be explained by the strongly increasing maximum rETR measured in this late phase of acclimation (Figure 7A). A moderate increase in expression levels of several genes encoding enzymes functioning in xanthophyll and Chl *a* metabolism was also observed during the late acclimation phase (Figure 1). The rise in expression levels of the genes connected to the formation of pigments is not reflected at the metabolic level, but might be explained by an increased demand of newly synthesized pigments in the HL cultures due to the shorter generation time at these light conditions (data not shown).

The vast majority of the genes predicted to be involved in carbon metabolism were affected by HL, and showed the strongest regulation during the late phase. Kroth *et al.* [42] suggested that a futile and energy demanding C4-like cycle might occur in *P. tricornutum* that possibly functions as a way to dissipate excess light energy. The results achieved in this experiment support a light regulated carbon metabolism; however, the complexity of the proposed model and the uncertainties connected to the localization of the proteins involved make interpretation of single gene regulation difficult. The regulation mechanisms of the Calvin cycle in diatoms are largely unknown. The modest regulation of a few genes believed to be involved in the Calvin cycle does not bring greater insight into this question, especially since most genes of the Calvin cycle are also involved in other pathways.

The late acclimation phase is recognized by low levels of light-harvesting pigments (Fuco and Chl *a*+*c*) occurring together with a pronounced increase in the photosynthetic capacity compared to the LL acclimated cultures (Figure 7). These measurements indicate that although the light-harvesting machinery has been downsized during the acclimation period, the adjustments made at the transcriptional and metabolic levels facilitate highly effective photosynthesis in HL-acclimated diatoms.

## Materials and Methods

An axenic culture of *P. tricornutum* Bohlin clone Pt 1 8.6 (CCMP632) was obtained from the culture collection of the Provasoli-Guillard National Center for Culture of Marine Phytoplankton, Bigelow Laboratory for Ocean Sciences, USA. Cultures were grown in f/2 medium [58] made with 0.2 µm-filtered and autoclaved local seawater supplemented with f/2 vitamins and inorganic nutrients [58], filter sterilized and added after autoclaving. Cultures were incubated at 15°C under cool white fluorescent light at scalar irradiance (E_PAR_) of approximately 35 µmol m^−2^s^−1^ (LL) on a rotary table in continuous light (control conditions), and were kept in exponential growth phase under these conditions for 3 weeks to ensure that all cells were acclimated. Scalar irradiance (Photosynthetic Active Radiance, 400–700 nm) in culture flasks was measured with a Biospherical QSL-100 irradiance sensor (San Diego, US). According to the growth curve based on cell counting and *in vivo* Chl *a* fluorescence with and without addition of DCMU (3-(3,4-dichlorophenyl)-1,1-dimethylurea), the cells divided once a day under these conditions. Sterility was monitored by occasional inoculation into peptone-enriched f/2 medium to check for bacterial growth [59]. Cells for the experiments were grown in batch cultures, and growth was monitored by cell counting using a Bürker-Türk counting chamber, counting 4–500 cells per volume-unity. The cells were first grown axenically in a 10-litre batch culture to reach an approximate density of 10^6^ cells mL^−1^, then volumes of 250 ml were transferred to 75 cm^2^ sterile Falcon polystyrene flasks to reach cell densities of 0.15–1.0×10^6^ cells mL^−1^ on the day of the experiment. The cultures were transferred to E_PAR_ irradiance conditions of 500 µmol m^−2^s^−1^ (HL = high light) and sampled at incubation times of 0.5 h, 3 h, 6 h, 12 h, 24 h and 48 h. In addition, LL control cultures were kept for each of the HL exposures and parallels. Three biological replica and two parallels for each treatment and control culture were harvested (6 samples) to ensure statistical validation. The two parallels for each of the biological replicas destined to be used for isolation of RNA were merged during harvesting to get enough starting material for the microarray analyses. The maximum cell density of 1.0×10^6^ cells mL^−1^ was chosen and carefully monitored and diluted to minimize effects like intercellular shading, rapid depletion of nutrients and increase in pH above 9. Material from the same cell cultures were utilized in the different analyses described below.

### RNA isolation and processing

Diatom cultures were centrifuged at 4000 g for 10 min at 15°C. The supernatant was removed and the cell pellet was resuspended in 1 ml f/2 medium. The suspension was transferred to 2 ml tubes and centrifuged at 18000 g for 1 min at 4°C. Supernatants were removed and the cell pellets were flash frozen in liquid nitrogen and stored at −80°C. Precooled (−80°C) 5 mm stainless steel beads (QIAGEN) were added to the tubes with frozen cell pellets, and the samples were mechanically disrupted and homogenized using the TissueLyser system (QIAGEN). Disruption was carried out for 2×2 min at 25 Hz. The samples were placed in a precooled (−80°C) adapter set for the first shaking step. Before the second shaking step, the samples were transferred to a room temperate adapter set and 0.5 ml lysis buffer (Spectrum^TM^ Plant Total RNA kit, Sigma-Aldrich) was added to each tube. Total RNA was isolated from the homogenized lysate using the Spectrum^TM^ Plant Total RNA kit (Sigma-Aldrich). On-column digestion of DNA with DNase I (QIAGEN) was included for all RNA preparations. The concentration of the RNA was determined by measuring the absorbance at 260 nm with the NanoDrop ND-1000 Spectrophotometer (NanoDrop Technologies), and the purity of the RNA was estimated by the OD_260_/OD_280_ nm absorption ratio (all ratios were >2.0). The integrity of the RNA was verified by denaturing agarose gel electrophoresis and ethidium bromide staining following the protocol described in the RNeasy Mini handbook (QIAGEN).

### DNA microarray experiments

Total RNA was isolated from three biological replicas of diatoms cultured under LL and HL conditions at time points 0.5 h, 3 h, 6 h, 12 h, 24 h and 48 h. The RNA (0.36–1 µg) was reverse transcribed, amplified and labelled using the Quick Amp Labelling Kit, Two-Color (Agilent p/n 5190-0444). Hybridization was performed with the Gene Expression Hybridization Kit (Agilent p/n 5188-5242). 825 ng cRNA from HL exposed cells were mixed with 825 ng cRNA from low light acclimated cells from the corresponding time point. The cRNA mixture was fragmented and hybridized on 4x44K *Phaeodactylum tricornutum* whole-genome 60-mer oligonucleotide microarrays (Agilent Technologies) in a rotary oven (10 rpm, 65°C, 17 h). The slides were washed with Gene Expression Wash Buffer 1 (Agilent p/n 5188-5325), Gene Expression Wash Buffer 2 (Agilent p/n 5188-5326), acetonitrile (VWR International) and Stabilization and Drying Solution (Agilent Technologies) according to the manufacturer's instructions. The slides were scanned at 5 µm resolution on an Agilent DNA microarray scanner (Agilent Technologies). The resulting images were processed using GenePix 5.1 software (Axon Instruments, Union City, USA).

#### Statistical analysis

The GenePix processed data were filtered to remove spots that had been flagged ‘Absent’, ‘Not Found’ or ‘Bad’, by the GenePix program or by manual flagging. Spots with more than 50% saturated pixels, or had median foreground intensity less than the local median background intensity were excluded from the analysis. No background subtraction was performed. For making statistical inference about differentially regulated genes the limma package [60] and the R statistical data analysis program package (R 2.7.1) was used. The limma approach is based on fitting a linear model to the expression data of each probe on a microarray. In this experiment each time point consists of 3 sets of biological replicates (test and control samples) which are dye swapped and adjusted for probe-specific dye effects. Due to some scale differences in the responses between samples, identified as differences in spread of M-values between the arrays, scale normalization between the arrays was performed. The Benjamini and Hochberg's method to control the false discovery rate (fdr) was used to identify differentially regulated genes [61]. Genes with adjusted P-values less than 0.05 were regarded as statistical significantly differentially expressed. If a gene was identified by less than 3 spots it was excluded from the result tables. Genes are represented by 1–5 different probes on each microarray. Expression ratios discussed in the text are an average of values obtained from all probes representing the genes in question. Supplementary information on the *P. tricornutum* genes are given in Table S1. The study is MIAME compliant. Raw data has been deposited in GEO (accession GSE 17237).

### Quantitative real-time PCR

A two-step quantitative real-time PCR (qRT-PCR) was performed on total RNA isolated from three biological replicas of diatom cultures grown in LL and HL at time points 0.5 h, 3 h, 12 h and 24 h. Reverse transcription of the RNA was performed with the PrimeScript^TM^ 1st strand cDNA Synthesis Kit (TaKaRa) following the recommended protocol for synthesis of real-time PCR template using random primers. 300 ng of total RNA was used in each reaction. 20 µl qRT-PCR mixtures were prepared containing forward and reverse primers listed in Supplementary Table S2, with a final concentration of 0.5 µM each, 5 µl cDNA template diluted 1:10 and 2x LightCycler® 480 SYBR Green I Master mix (Roche). The qRT-PCR reactions were run in a LightCycler® 480 Multiwell Plate 96 (Roche) in a LightCycler 480 instrument (Roche). No template controls, where the cDNA template was replaced with PCR-grade water, were included in each run to ensure that no reagents were contaminated with DNA. To detect the level of genomic DNA still present in the 24 RNA samples after the DNase I treatment, qRT-PCR was performed using 7.5 ng of isolated RNA as template, and three different primer pairs listed in Supplementary Table S2. The PCR parameters were programmed according to the manufacturer's instructions for a LightCycler 480 System PCR run with the LightCycler® 480 SYBR Green I Master: 5 min preincubation at 95°C, followed by 35 cycles with 10 s at 95°C, 10 s at 55°C and 10 s at 72°C. After 35 cycles the specificity of the amplified PCR products was tested by heating from 65°C up to 95°C with a ramp rate of 2.2°C/s, resulting in melting curves. The Second Derivative Maximum Method of the LightCycler 480 software was used to identify the crossing points (CPs) of the samples. LinRegPCR software [62] was used to determine the PCR efficiency for each sample. The primer set efficiency was determined by calculating the mean of the efficiency values obtained from the individual samples. Relative expression ratios of the target genes normalized to a reference gene encoding a putative hiv-1 rev binding protein (Phatr2_42776) were calculated using the REST 2005 software [63]. The primer efficiencies determined by LinRegPCR were included in the calculations. REST 2005 was also used to test significance of the expression ratio results of the investigated transcripts by a Pair Wise Fixed Reallocation Randomisation Test. The gene encoding the hiv-1 rev binding protein that was chosen to function as a reference gene is represented by five different probes on the microarray and showed no response to the HL treatment at any time point.

### Pigment analysis

HPLC pigment analyses were performed as described in Rodriguez *et al.* [25] using a Hewlett-Packard HPLC 1100 Series system. The pigment values from the HPLC analyses were calculated as femtogram (fg) pigment per cell. De-epoxidation state index (DES) was calculated as in Ruban *et al.* [14]: DES = (DT)/((DD)+(DT)).

### Variable Chl fluorescence

Variable Chl *a* fluorescence was measured using a PhytoPAM (System I, Walz, Germany), equipped with a sensitive Photomultiplier-Detector (PM-101P, Walz). Fluorescence was excited by a weak and nonactinic modulated light supplied by a LED (light emitting diode, Array-Cone PHYTO-ML, Walz, Germany) and a saturating flash by a strong red LED (>2000 µmol m^−2^ s^−1^, Actinic LED-Array-Cone PHYTO-AL, Walz) to ensure that all reaction centres of PSII were closed during the flash period. The instrument light source excites fluorescence at four different wavelengths; however, only results from 665 nm excitation were used. Nomenclature of van Kooten and Snell [64] was used. The minimum (F_0_) and maximum fluorescence (F_m_) was measured at the end of a dark-acclimation period (3 min) and the maximum quantum yield (the PSII efficiency) was calculated from F_v_/F_m_, where F_v_ is the variable part of fluorescence emission and equal to F_m_–F_0_. The photosynthesis *vs.* irradiance (P *vs.* E) relationship was obtained by exposing the samples (after the 3 min dark-acclimation) to 12 step-wise increasing irradiances (1 and 1200 µmol photons m^−2^ s^−1^) at intervals of 30 s each. The operational quantum yield of PSII (Φ_PSII_) was calculated from the steady-state fluorescence (F_s_) at each irradiance and the maximum fluorescence measured after a saturation pulse (F_m_′) at the end of each irradiance interval, from (F_m_′–F_s_)/F_m_′ [6]. The relative electron transport rates (rETR) was then calculated from multiplying Φ_PSII_ with the incubation irradiance, and the relationship was fitted using the build-in hyperbolic fitting routine in the PhytoWin software package (ver. 2.00a) to determine the maximum light utilization coefficient (α) and the maximum rETR. The light saturation index was calculated from rETR divided by α [65], [66]. A Peltier cell (US-T/S, Walz) kept the temperature constant (±0.2°C) during incubations.

## Supporting Information

Figure S1 Regulation pattern of eight chloroplast-encoded genes during the HL acclimation period. Expression ratios are investigated by both microarray analyses (A) and qRT-PCR (B). The color code indicates expression values and squares with a diagonal line inside indicate genes with an expression ratio (log2 transformed) greater than +/− 0.5 that are not significantly regulated. The scale on the right represents gene expression ratio values, log2 transformed. The abbreviations used are NA: not assessed; ChlI: subunit I of magnesium chelatase; FtsH: Filamentation temperature sensitive H; Psa: PSI proteins; Psb: PSII proteins; RbcS: ribulose-bisphosphate carboxylase oxygenase small subunit; RbcL: ribulose-bisphosphate carboxylase oxygenase large subunit.
(0.69 MB EPS)

Table S1 Information on P. tricornutum genes discussed in the text. Parameters given are the protein identification number (ID), chromosome location, best NCBI hit (BLASTP search) outside the Bacillariophyceae, the identity/similarity with respect to the corresponding P. tricornutum protein and respective NCBI accession numbers.
(0.13 MB XLS)

Table S2 Primers used for quantitative real-time PCR.
(0.03 MB XLS)

## References

[1] Field CB, Behrenfeld MJ, Randerson JT, Falkowski P (1998). Primary production of the biosphere: integrating terrestrial and oceanic components.. Science.

[2] Long S, Humphries S, Falkowski P (1994). Photoinhibition of photosynthesis in nature.. Annu Rev Plant Physiol Plant Mol Biol.

[3] Raven JA, Geider RJ, Larkum AW, Douglas SE, Raven JA, (2003). Adaptation, acclimation and regulation in algal photosynthesis.. Photosynthesis in algae.

[4] Armbrust V, Berges JA, Bowler C, Green B, Martinez D (2004). The genome of the diatom *Thalassiosira pseudonana*: ecology, evolution, and metabolism.. Science.

[5] Bowler C, Allen AE, Badger JH, Grimwood J, Jabbari K (2008). The *Phaeodactylum* genome reveals the evolutionary history of diatom genomes.. Nature.

[6] Genty B, Briantais JM, Baker NR (1989). The relationship between the quantum yield of photosynthetic electron-transport and quenching of chlorophyll fluorescence.. Biochim Biophys Acta.

[7] Falkowski PG, Chen YB, Green BR, Parson WW, (2003). Photoacclimation of light harvesting systems in eukaryotic algae.. Light harvesting antennas in photosynthesis.

[8] Müller P, Li XP, Niyogi KK (2001). Non-photochemical quenching. A response to excess light energy.. Plant Physiol.

[9] Horton P, Ruban AV, Walters RG (1996). Regulation of light harvesting in green plants.. Ann Rev Plant Physiol Plant Mol Biol.

[10] Gilmore AM (1997). Mechanistic aspects of xanthophyll cycle-dependent photoprotection in higher plant chloroplasts and leaves.. Physiol Planta.

[11] Lavaud J, Rousseau B, Etienne AL (2004). General features of photoprotection by energy dissipation in planktonic diatoms (Bacillariophyceae).. J Phycol.

[12] Johnsen G, Prézelin BB, Jovine RVM (1997). Fluorescence excitation spectra and light utilization in two red tide dinoflagellates.. Limnol Oceanogr.

[13] Brunet C, Johnsen G, Lavaud J, Roy S, Roy S, Egeland E, Johnsen G, Llewellyn C, (2009). Pigments and photoacclimation processes.. Pigments in Oceanography.

[14] Ruban AV, Lavaud J, Rousseau B, Guglielmi G, Horton P, Etienne AL (2004). The super-excess energy dissipation in diatom algae: comparative analysis with higher plants.. Photosynth Res.

[15] Owens TG (1986). Light-harvesting function in the diatom *Phaeodactylum tricornutum*. II. Distribution of excitation energy between the photosystems.. Plant Physiol.

[16] Horton P, Ruban AV (1992). Regulation of photosystem II.. Photosynth Res.

[17] Barber J (1995). Molecular basis of the vulnerability of photosystem-II to damage by light.. Aust J Plant Physiol.

[18] Niyogi KK (1999). Photoprotection revisited: genetic and molecular approaches.. Annu Rev Plant Physiol Lant Mol Biol.

[19] Adir N, Zer H, Shochat S, Ohad I (2003). Photoinhibition – a historical perspective.. Photosynth Res.

[20] Ohad I, Adir N, Koike H, Kyle DJ, Inoue Y (1990). Mechanism of photoinhibition in vivo. A reversible light-induced conformational change of reaction center II is related to an irreversible modification of the D1 protein.. J Biol Chem.

[21] Yamamoto Y, Aminaka R, Yoshioka M, Khatoon M, Komayama K (2008). Quality control of photosystem II: impact of light and heat stresses.. Photosynth Res.

[22] Shapira M, Lers A, Heifetz PH, Irihimovitz V, Osmond CB (1997). Differential regulation of chloroplast gene expression in *Chlamydomonas reinhardtii* during photoacclimation: light stress transiently suppresses synthesis of the Rubisco LSU protein while enhancing synthesis of the PS II D1 protein.. Plant Mol Biol.

[23] Hihara Y, Kamei A, Kanehisa M, Kaplan A, Ikeuchi M (2001). DNA microarray analysis of cyanobacterial gene expression during acclimation to high light.. Plant Cell.

[24] Stolte W, Kraay GW, Noordeloos AAM, Riegman R (2000). Genetic and physiological variation in pigment composition of *Emiliania huxleyi* (Prymnesiophyceae) and the potential use of its pigment ratios as a quantitative physiological marker.. J Phycol.

[25] Rodriguez F, Chauton M, Johnsen G, Andresen K, Olsen LM, Zapata M (2006). Photoacclimation in phytoplankton: implications for biomass estimates, pigment functionality and chemotaxonomy.. Mar Biol.

[26] Perry MJ, Talbot MC, Alberte RS (1981). Photoadaptation in marine phytoplankton: Response of the photosynthetic unit.. Mar Biol.

[27] Clayton RK, Wang RT (1971). Photochemical reaction centers from Rhodopseudomonas-spheroids..

[28] Nakamura K, Ogawa T, Shibata K (1976). Chlorophyll and peptide compositions in the two photosystems of marine green algae.. Biochim Biophys Acta.

[29] Schuster G, Lisitsky I, Klaff P (1999). Polyadenylation and degradation of mRNA in the chloroplast.. Plant Physiol.

[30] Tanaka R, Tanaka A (2007). Tetrapyrrole biosynthesis in higher plants.. Annu Rev Plant Biol.

[31] Wilhelm C, Büchel C, Fisahn J, Goss R, Jakob T (2006). The regulation of carbon and nutrient assimilation in diatoms is significantly different from green algae.. Protist.

[32] Coesel S, Obornik M, Varela J, Falciatore A, Bowler C (2008). Evolutionary origins and functions of the carotenoid biosynthetic pathway in marine diatoms.. PLoS ONE.

[33] Zhu SH, Green B, Allen JF, Gantt E, Golbeck JH, (2008). Light-harvesting and photoprotection in diatoms: identification and expression of L818-like proteins in photosynthesis.. Energy from the Sun, 14th International Congress on Photosynthesis.

[34] Nelson N, Yocum CF (2006). Structure and function of photosystems I and II.. Annu Rev Plant Biol.

[35] Oudot-Le Secq MP, Grimwood J, Shapiro H, Armbrust EV, Bowler C, Green BR (2007). Chloroplast genomes of the diatoms *Phaeodactylum tricornutum* and *Thalassiosira pseudonana*: comparison with other plastid genomes of the red lineage.. Mol Genet Genomics.

[36] Enami I, Okumura A, Nagao R, Suzuki T, Iwai M, Shen JR (2008). Structures and functions of the extrinsic proteins of photosystem II from different species.. Photosynth Res.

[37] Chen H, Zhang D, Guo J, Wu H, Jin M (2006). A Psb27 homologue in *Arabidopsis thaliana* is required for efficient repair of photodamaged photosystem II.. Plant Mol Biol.

[38] Plücken H, Müller B, Grohmann D, Westhoff P, Eichacker LA (2002). The HCF136 protein is essential for assembly of the photosystem II reaction center in *Arabidopsis thaliana*.. FEBS lett.

[39] Roose JL, Pakrasi HB (2008). The Psb27 protein facilitates manganese cluster assembly in photosystem II.. J Biol Chem.

[40] Komenda J, Nickelsen J, Tichý M, Prášil O, Eichacker LA (2008). The cyanobacterial homologue of HCF136/YCF48 is a component of an early photosystem II assembly complex and is important for both the efficient assembly and repair of photosystem II in Synechocyctis sp. PCC 6803.. J Biol Chem.

[41] Jensen PE, Bassi R, Boekema EJ, Dekker JP, Jansson S (2007). Structure, function and regulation of plant photosystem I.. Biochim Biophys Acta.

[42] Kroth PG, Chiovitti A, Gruber A, Martin-Jezequel V, Mock T (2008). A model for carbohydrate metabolism in the diatom *Phaeodactylum tricornutum* deduced from comparative whole genome analysis.. PLoS ONE.

[43] Krieger-Liszkay A, Fufezan C, Trebst A (2008). Singlet oxygen production in photosystem II and related protection mechanism.. Photosynth Res.

[44] Li Y, Wang Z, Sun X, Tang K (2008). Current opinions on the functions of tocopherol based on the genetic manipulation of tocopherol biosynthesis in plants.. J Integr Plant Biol.

[45] Lamkemeyer P, Laxa M, Collin V, Li W, Finkemeier I (2006). Peroxiredoxin Q of *Arabidopsis thaliana* is attached to the thylakoids and functions in context of photosynthesis.. Plant J.

[46] Meyer Y, Siala W, Bashandy T, Riondet C, Vignols F (2008). Glutaredoxins and thioredoxins in plants.. Biochim Biophys Acta.

[47] Schürmann P, Buchanan BB (2008). The ferredoxin/thioredoxin system of oxygenic photosynthesis.. Antioxid Redox Signal.

[48] Wang F, Zhong NQ, Gao P, Wang GL, Wang HY (2008). SsTypA1, a chloroplast-specific TypA/BipA-type GTPase from the halophytic plant *Suaeda salsa*, plays a role in oxidative stress tolerance.. Plant Cell Environ.

[49] Brown JS (1988). Photosynthetic pigment organization in diatoms (Bacillariophyceae).. J Phycol.

[50] Oeltjen A, Krumbein WE, Rhiel E (2002). Investigations on transcript sizes, steady state mRNA concentrations and diurnal expression of genes encoding fucoxanthin chlorophyll a/c light harvesting polypeptides in the centric diatom *Cyclotella cryptica*.. Plant Biol.

[51] Mock T, Valentin K (2004). Photosynthesis and cold acclimation: molecular evidence from a polar diatom.. J Phycol.

[52] Lohr M, Wilhelm C (1999). Algae displaying the diadinoxanthin cycle also possess the violaxanthin cycle.. Proc Natl Acad Sci U S A.

[53] Lohr M, Wilhelm C (2001). Xanthophyll synthesis in diatoms: quantification of putative intermediates and comparison of pigment conversion kinetics with rate constants derived from a model.. Planta.

[54] Savard F, Richard C, Guertin M (1996). The *Chlamydomonas reinhardtii* LI818 gene represents a distant relative of the cabI/II genes that is regulated during the cell cycle and in response to illumination.. Plant Mol Biol.

[55] Richard C, Ouellet H, Guertin M (2000). Characterization of the LI818 polypeptide from the green unicellular alga *Chlamydomonas reinhardtii*.. Plant Mol Biol.

[56] Zhirong L, Wakao S, Fischer BB, Niyogi KK (2009). Sensing and responding to excess light.. Annu Rev Plant Biol.

[57] Ballottari M, Dall'Osto L, Morosinotto T, Bassi R (2007). Contrasting behaviour of higher plant photosystem I and II antenna systems during acclimation.. J Biol Chem.

[58] Guillard RRL, Smith WL, Chanley MH, (1975). Culture of phytoplankton for feeding marine invertebrates.. Culture of marine invertebrate animals.

[59] Andersen RA, Morton SL, Sexton JP (1997). Provasoli-Guillard National Center for culture of marine phytoplankton 1997 list of strains.. J Phycol.

[60] Smyth GK (2004). Linear models and empirical Bayes methods for assessing differential expression in microarray experiments..

[61] Benjamini Y, Hochberg Y (1995). Controlling the false discovery rate: a practical and powerful approach to multiple testing.. J R Statist Soc B.

[62] Ramakers C, Ruijter JM, Deprez RH, Moorman AFM (2003). Assumption-free analysis of quantitative real-time polymerase chain reaction (PCR) data.. Neurosci Lett.

[63] Pfaffl MW, Horgan GW, Dempfle L (2002). Relative expression software tool (REST©) for group-wise comparison and statistical analysis of relative expression results in real-time PCR.. Nucleic Acids Res.

[64] Van Kooten O, Snell JFH (1990). The use of chlorophyll fluorescence nomenclature in plant stress physiology.. Photosynth Res.

[65] Hancke K, Hancke TB, Olsen LM, Johnsen G, Glud R (2008). Temperature effects on microalgae photosynthesis-light responses measured by O_2_ production, pulse-amplitude-modulated fluorescence and ^14^C assimilation.. J Phycol.

[66] Sakshaug E, Bricaud A, Dandonneau Y, Falkowski PG, Kiefer DA (1997). Parameters of photosynthesis: definitions, theory and interpretation of results.. J Plankton Res.

